# Psychometric properties of the FACES IV package for Spanish adolescents

**DOI:** 10.1186/s41155-022-00222-2

**Published:** 2022-06-20

**Authors:** María I. Vegas, Manuel Mateos-Agut, Pedro J. Pineda-Otaola, Carlota Sebastián-Vega

**Affiliations:** 1 Universidad de Burgos, C/ Alfonso XI, s/n, 09007 Burgos, Spain; 2 Day Hospital, Psychiatry Service, Burgos Universitary Hospital, Avda. Islas Baleares 3, 09006 Burgos, Spain; 3 Technical Professor of Community Services, Junta de Castilla y León, Burgos, Spain; 4 Social Services of Aranda de Duero (Burgos), Aranda de Duero, Burgos Spain

**Keywords:** FACES IV, Circumplex model, Adolescence, Family Communication, Family Satisfaction, Cohesion, Flexibility, Family functioning, Validation, Family assessment

## Abstract

The family plays an essential role in the life of an adolescent. Hence, an acceptable understanding and an evaluation of family functioning is fundamental for effective interventions with adolescents in the psychological, social, and educational fields. The main purpose of this study is to examine the psychometric properties of the Family Adaptability and Cohesion Evaluation Scale (FACES IV), the Family Communication Scale (FCS), and the Family Satisfaction Scale (FSS), for assessing the family functioning of Spanish adolescents. The sample was comprised of 1187 adolescents between 14 -18 years old (49.96% boys and 50.04% girls; M = 16.17; SD = 1.31) from Castile and Leon (Spain), selected from 23 educational centers, 10 university degree courses, and 18 specific juvenile centers for adolescents with either family or behavioral problems. The scales of Balanced Cohesion, Balanced Flexibility and Disengaged showed good convergent validity, while Enmeshed, Rigid, and Chaotic did not. For this reason some items were removed, obtaining a shortened version of FACES IV, that demonstrated acceptable reliability, and good convergent and predictive validity. The FCS and FSS scales yielded excellent psychometric properties. The results confirmed the factorial structure of the FACES IV, its transcultural applicability, and its validity for different ages. The hypotheses of the circumplex model were confirmed, except for the dysfunctionality of two scales, Enmeshed and Rigid, that contrary to what was expected, showed positive correlations with Family Communication, Family Satisfaction, Balanced Cohesion, and Balanced Flexibility. In brief, our results present the FACES IV package as a useful instrument for the assessment of family functioning of Spanish adolescents. Future studies will be necessary to confirm the trend observed for the two aforementioned scales among adolescents.

## Introduction

The family plays an essential role in the life of an adolescent. It is the principal source of protection, security, emotional support, and socialization, and the model for the transfer of norms, values and beliefs, and for the moral development of the adolescent (Bhugra & Fiorillo, 2012; Minuchin, 2009; Samper et al., 2006; UN, 2016). Families are changing nowadays and many new problems have emerged in the family scene over recent decades, with direct impacts on the perceptions, the feelings, and the conduct of adolescents. Hence, an acceptable understanding and an evaluation of family functioning is fundamental for effective interventions with adolescents in the psychological, social, and educational fields.

Over 200 concepts have been used in family development theory, in order to try to assess the principal aspects of couple and family functioning. In the late 1970s, these concepts were clustered into three main dimensions (Olson et al., 2019), giving rise to the Circumplex Model of Family and Marital Systems (Olson et al., 1979). Based on this model, the *Family Adaptability and Cohesion Evaluation Scale* (FACES) was designed. This is one of the most important questionnaires at an international level for the assessment of family functioning, because of its theoretical foundations, and its applicability to clinical scenarios and investigations with families (Hamilton & Carr, 2016; Jiménez et al., 2017; Olson, 2008; White & Klein, 2008). This model incorporates the systemic theory and the theory of family development (Olson & Gorall, 2003) and has been used in over 1,200 studies (Olson et al., 2019). Sanderson et al. (2009) pointed to the FACES questionnaire as the third measure of family functioning that is most frequently used in investigations on family and couple therapy.

According to the circumplex model, the three dimensions that are considered crucial to understand family functioning are Cohesion, Flexibility, and Communication (Barnes & Olson, 1985; Olson, 2011; Olson & Gorall, 2003; Olson et al., 1979). Cohesion is defined as “the emotional bonding that family members have toward one another” (Olson et al., 2019, p. 201). Flexibility is “the amount of change in its leadership, roles relationships, and relationship rules*”* (Olson et al., 2019, p. 202). Communication is considered as a facilitating dimension of the other two and is defined as “the positive communication skills utilized in the couple or family system” (Olson, 2011, p.65).

The principal hypotheses on family functioning arising from the circumplex model are that partners and families with balanced levels of Cohesion and Flexibility will function more effectively than those with extreme values in both dimensions and that positive communication skills will permit the balanced systems to modify their levels of Cohesion and Flexibility and will, therefore, give rise to systems with healthier family functioning (Olson & Gorall, 2003; Olson et al., 1979, 2019).

It may be inferred from the above definitions that the dimensions of Cohesion and Flexibility have a curvilinear relation with family functioning, because either very high or very low values are problematic or dysfunctional. The same is not so with Communication, considered as a linear dimension, as the higher the communicative value that is achieved, the better the system will function (Olson et al., 1979, 2019).

The level of positive communication within the family is measured through the *Family Communication Scale* (FCS) (Barnes & Olson, 1985), whereas Cohesion and Flexibility are measured through the questionnaire *Family Adaptability and Cohesion Evaluation Scale* (FACES). Over the past four decades, there have been numerous versions of the circumplex model, the last of which, FACES IV, validated by Olson in 2011, presented the possibility of covering the central and the extreme parts of Cohesion and Flexibility through six scales. The dimension Cohesion includes three variables, *Balanced Cohesion, Disengaged*, *and Enmeshed*, while the dimension Flexibility includes *Balanced Flexibility, Rigid*, *and Chaotic*. The variables *Balanced Cohesion* and *Balanced Flexibility* function as balanced scales and cover the intermediate parts of each dimension, while the variables *Disengaged, Enmeshed, Rigid, and Chaotic* are the extreme or unbalanced scales (Olson, 2011) and cover the extremely high or extremely low levels of both dimensions.

These scales that cover the three basic constructs of the circumplex model were later on supplemented with the *Family Satisfaction Scale* (FSS) by Olson (Olson, 2000; Olson et al., 2019), a form of measuring satisfaction through the three principal dimensions of the circumplex model.

The FACES IV version has been validated for the adult population in various European countries (Baiocco et al., 2013; Gomes et al., 2017; Koutra et al., 2012; Margasiński, 2015; Mirnics et al., 2010; Pereira & Teixeira, 2013; Sequeira et al., 2021) and, likewise, in Spain by Martínez-Pampliega et al. (2017). The FCS and FSS scales have also been validated in Spain (Sanz et al., 2002) and other countries (Costa-Ball & Cracco, 2021; Cracco & Costa-Ball, 2019; Gomes et al., 2017; Koutra et al., 2012; Pereira & Teixeira, 2013; Sequeira et al., 2021).

Everri et al. (2020) insisted on the utility of the FACES IV scales for adolescents, because it provides more information on understanding the parent–offspring relations. However, none of the FACES IV Package scales has been validated for the population of Spanish adolescents and a few validations have been conducted for adolescents worldwide. We consider that an *ex profeso* validation of the FACES IV Package for adolescents is necessary, because adolescents have very specific family needs and present peculiar features, which requires a differentiated study of family functioning. In fact, the authors of international studies completed with adolescents using FACES IV (Baiocco et al., 2013; Desautels et al., 2016; Everri et al., 2015, 2016, 2020; Gouveia-Pereira et al., 2020; Sebokova et al., 2016) all agree that the tool has to be validated with adolescents, due to the specific characteristics of this population. “When non-adult samples are concerned, additional aspects need to be taken in consideration” (Everri et al., 2020, p. 2508). In the Canadian study, the results suggested a degree of fine-tuning in the use of the version with adolescents, despite Olson and Gorall’s recommendations that the FACES IV may be used with both adults and adolescents (Desautels et al., 2016, p. 110).

On the other hand, extremely low levels of Flexibility (Rigid) or extremely high levels of Cohesion (Enmeshed) are hypothesized as problematic for individuals and relational development in general, according to the predictions of Olson’s circumplex model (Olson et al., 2019). Nevertheless, these standards are not necessarily evident among adolescents: in their studies conducted with adolescents, Baiocco et al. (2013) and Everri et al., (2015, 2016), found positive correlations between Rigid and the balanced variables, Cohesion and Flexibility, and between Rigidity and Enmeshed. In the studies of Everri et al*.*, Rigidity consistently emerged as an indicator of adaptive family functioning, and an association was found between Rigidity and parental monitoring, as well as between Rigidity and family responsibility. The participants ‘‘might have interpreted rigidity as a protective emotional bond related to more general parental engagement, *e.g.*, awareness of their children’s activities, friends and interests’’ (Everri et al., 2015, p. 3064). In the work of Everri et al. (2016), Rigidity was also positively associated with family satisfaction.

These works on adolescents, where consideration is given to adolescents’ points of view, raise a controversy over the dysfunctionality of the variables Rigidity and Enmeshed, pointing to the need for a specific study of both variables among adolescents.

The first objective of the present study is, therefore, to test the validity and the reliability of the *Family Communication Scale*, the *Family Satisfaction Scale*, and the FACES IV scales among Spanish adolescents.

A second objective is to test whether the variables Rigidity and Enmeshed correlate negatively with family communication, family satisfaction, and the balanced variables of FACES IV, in our sample of adolescents.

## Method

### Participants

A total of 1,187 adolescents (49.96% boys and 50.04% girls), aged between 14 and 18 years old (M = 16.17; SD = 1.31), participated in the study. The sample had the following distribution by age: 14 years old = 14.8%; 15 years old = 16.3%, 16 years old = 22.8%; 17 years old = 28.8%; 18 years old = 17.3%. The adolescents were selected from 51 centers, grouped into three large clusters: a) 23 educational centers within the province of Burgos –Spain–, including Obligatory Secondary Education (35.5%), pre-university studies –Baccalaureate– (28.8%), Intermediate Level Vocational Training (3.6%), and Basic Professional Training (15.7%); b) 10 university degree courses at Burgos University –Spain– (6.9%), and c) 18 juvenile facilities of Castile and Leon –Spain–, including child protection centers and centers for adolescents with family problems (4.1%), juvenile centers for youth with behavioral and drug dependency problems (1.7%), and juvenile offenders (3.8%).

In all, 77.9% lived in a nuclear family, 9.9% lived only with their mother, 2.8% lived only with their father, 2.9% had shared parenting, and 5.1% belonged to a reconstituted family. 1.4% lived in other family arrangements different from the above.

All participants were Spanish.

### Procedure

The sampling at the non-university educational centers was completed in two stages: stratified sampling (center and educational level) and sampling by blocks (each group/class). All centers from the province of Burgos were classified into 12 levels, considering the educational level of the center (a: pre-University; b: Obligatory Secondary; c: Intermediate Level Vocational Training; d: Basic Professional Training) and the location and type of center (a-urban public; b-rural public; c-urban private). At each level, at least 5% of educational centers were selected and when a center refused to participate in the study, it was replaced by another with similar characteristics. The centers with fewer than 100 students were not included in the sample because of their scant representativeness. One group/class was selected for each center and educational level, in which we had the collaboration of a teacher, and the voluntary participation of all students from the class was requested. The questionnaire was administered in person by the teachers with instructions from the researchers.

Convenience sampling was used at the university centers. In this group, the questionnaire was completed on an individual basis outside the classroom.

All the juvenile centers in the province of Burgos were visited for the sampling at non-educational centers and participation was massive. At these centers, all the adolescents were invited to participate and the questionnaires were individually administered with the assistance of an educator.

Informed consent from either the youth or their legal guardians was obtained, ensuring the anonymity and the confidentiality of the data. The participation of all the adolescents in the study was voluntary. All procedures for this study were performed in accordance with the ethical standards of the University of Burgos research committee and with the Helsinki Declaration and its later amendments.

### Research instruments

The adolescents were asked to complete a self-administered questionnaire, composed of various socio-demographic questions and the FACES IV Package, which consisted of three questionnaires:

- the Spanish version of the FACES IV scale (Olson, 2011), validated for adults (Martínez-Pampliega et al., 2017). This questionnaire has 42 items, 7 items for each of their six variables: two balanced (*Cohesion and Flexibility*) and four unbalanced (*Disengaged, Enmeshed, Rigid, and Chaotic).* Each item could be answered on a 5-point Likert-type scale: 1 (*Strongly Disagree*) to 5 (*Strongly Agree*).
- the *Family Communication Scale* (FCS) (Barnes & Olson, 1985), with 10 items and responses on a 5-point Likert-type scale: 1 (*Strongly Disagree*) to 5 (*Strongly Agree*).
- the *Family Satisfaction Scale* (FSS) (Olson, 2000), with 10 items, with responses on a 5-point Likert-type scale: 1 (*Very Dissatisfied*) to 5 (*Very Satisfied*).

Both the FCS and the FSS have been validated in Spain for the adult population (Sanz et al., 2002). The Spanish versions were used for our study.

### Data analysis

First, a Confirmatory Factor Analysis (CFA) for all 42 items was performed, in order to validate the structure of the FACES IV constructs and to test their convergent validity. The following quality indices of the model were considered (Chen et al., 2008; Ferrando & Anguiano-Carrasco, 2010; Hair et al., 2010; Lloret-Segura et al., 2014; Malhotra, 2010; Montero & León, 2007; Xia & Yang, 2019): the minimum discrepancy or likelihood ratio Chi-square test divided by its degrees of freedom (CMIN/df), the Goodness of Fit Index (GFI), the Adjusted Goodness of Fit Index (AGFI), the Normed Fit Index (NFI), the Incremental Fit Index (IFI), the Comparative Fit Index (CFI), and the Root Mean Squared Error of Approximation (RMSEA). The Composite Reliability (CR), and the Average Variance Extracted (AVE) were used to complete the convergent validity. The values considered to guarantee the quality of goodness of fit indexes were: CMIN/df < 5; GFI > 0.95; AGFI > 0.80; NFI > 0.80; IFI > 0.85; CFI > 0.90; RMSEA < 0.05 (acceptable between 0.05 and 0.08), and a *p*-value > 0.05. CR should be greater than 0.7 and AVE greater than 0.5. A minimum value of 0.3 was required to maintain the factor loadings within each dimension (Hair et al., 2010). The *t*-student test was applied, to check that the factor loadings were significantly non-zero.

As an additional tool to establish the structure and the functioning of the 6 variables of FACES IV and both the FCS and the FSS scales, a Principal Components Analysis (PCA) with the Varimax rotation method was separately performed for each variable, in order to determine the unidimensionality of each construct (Hattie, 1985). The Kaiser–Meyer–Olkin (KMO) measure, the Bartlett’s test of sphericity (χ^2^), the Carmines and Zeller criterion (variance explained by each factor must be at least 40%), and the Kaiser criterion (eigenvalues should be greater than one) were used to determine the sampling adequacy of the PCA (Hair et al., 2010; Hattie, 1985; Lloret-Segura et al., 2014; Malhotra, 2010). A sufficiently high KMO value (higher values than 0.7 ensure sufficient data adequacy, higher than 0.8 a satisfactory fit, and below 0.5, an inadequate fit), and a *p*-value < 0.05 in Bartlett’s test of sphericity indicate the appropriateness of factor analysis.

Descriptive statistics for each scale were calculated: mean and standard deviation. The reliability analysis was performed using Cronbach’s alpha coefficients.

Finally, the inter-scale correlations were assessed with Pearson’s correlation coefficient and predictive validity through discriminant analysis, following the specific guidelines from Olson (2011).

For the statistical analyses, CFA was performed with the statistical software package IBM SPSS AMOS 16.0 and AMOS 23, and PCA, reliability, descriptive statistics and the Pearson correlations were calculated with IBM SPSS Statistics 22 software.

## Results

### Factor analysis of the FACES IV scales

A Confirmatory Factor Analysis (CFA) of the FACES IV model with the 42 items originally proposed by Olson (2011) was performed (see Table 1). The CFA showed 7 items with factor loadings below 0.3, specifically items 10, 22, 34, 29, 41, 12, and 30, corresponding to the scales Enmeshed, Rigid, and Chaotic.

**Table 1:** Confirmatory Factor Analysis (CFA) results for the 42 FACES IV items

Factor loadings for each item			Factor loadings for each item		
Item 1	Balanced Cohesion	.549	Item 38	Balanced Flexibility	.722
Item 7	Balanced Cohesion	.754	Item 32	Balanced Flexibility	.381
Item 13	Balanced Cohesion	.720	Item 26	Balanced Flexibility	.423
Item 19	Balanced Cohesion	.561	Item 20	Balanced Flexibility	.546
Item 25	Balanced Cohesion	.573	Item 14	Balanced Flexibility	.602
Item 31	Balanced Cohesion	.655	Item 8	Balanced Flexibility	.553
Item 37	Balanced Cohesion	.630	Item 2	Balanced Flexibility	.540
Item 40	Enmeshed	.323	Item 5	Rigid	.625
Item 34	Enmeshed	.096^a^	Item 11	Rigid	.556
Item 28	Enmeshed	.723	Item 17	Rigid	.644
Item 22	Enmeshed	.147^a^	Item 23	Rigid	.525
Item 16	Enmeshed	.519	Item 29	Rigid	.211^a^
Item 10	Enmeshed	.050^a^	Item 35	Rigid	.614
Item 4	Enmeshed	.631	Item 41	Rigid	.170^a^
Item 3	Disengaged	.614	Item 42	Chaotic	.697
Item 9	Disengaged	.688	Item 36	Chaotic	.511
Item 15	Disengaged	.436	Item 30	Chaotic	.113^a^
Item 21	Disengaged	.638	Item 24	Chaotic	.342
Item 27	Disengaged	.646	Item 18	Chaotic	.674
Item 33	Disengaged	.334	Item 12	Chaotic	.050^a^
Item 39	Disengaged	.392	Item 6	Chaotic	.584

^a^items with factor loadings below 0.3

On the other hand, the CFA fit indices yielded no acceptable fit, as the criteria of some of the indices were not at an optimum level of quality (Hair et al., 2010): GFI = 0.87 (yet should be > 0.95); NFI = 0.769 (yet should be > 0.80); IFI = 0.798 (yet should be > 0.85); CFI = 0.811 (yet should be > 0.90); *p*-value of the RMSEA = 0.006 (yet should be > 0.05).

As a complementary measure to establish the structure and the functioning of the six variables of FACES IV in the Spanish sample of adolescents, we carried out a Principal Component Analysis (PCA) on each of the six theoretical constructs proposed by Olson, with the aim of confirming the unidimensionality of each construct. A global Exploratory Factor Analysis (EFA) on the 42 items was not conducted, in search of a new underlying structure of the data, because the six-factor structure has been confirmed by all the validation studies on FACES IV over the past 15 years, both among the adult and adolescent population, with either the original version or the abridged versions of FACES IV (Baiocco et al., 2013; Costa et al., 2013; Desautels et al., 2016; Everri et al., 2020; Gomes et al., 2017; Gouveia-Pereira et al., 2020; Koutra et al., 2012; Martínez-Pampliega et al., 2017; Pereira & Teixeira, 2013; Rivero et al., 2010; Sebokova et al., 2016).

We therefore performed a PCA with Varimax rotation method on each of the six constructs (variables), using the Kaiser criterion (the eigenvalues-greater-than-1 rule). The results are presented in Table 2.

**Table 2:** Principal components analysis with Varimax rotation on each of the FACES IV constructs

	Balanced Cohesion	Balanced Flexibility	Disengaged	Enmeshed		Rigid		Chaotic	
	Item 1	Item 2	Item 3	Item 4	Item 10	Item 5	Item 29	Item 6	Item 12
	Item 7	Item 8	Item 9	Item 16	Item 22	Item 11	Item 41	Item 18	Item 24
	Item 13	Item 14	Item 15	Item 28	Item 34	Item 17		Item 36	Item 30
	Item 19	Item 20	Item 21		Item 40	Item 23		Item 42	
	Item 25	Item 26	Item 27			Item 35			
	Item 31	Item 32	Item 33						
	Item 37	Item 38	Item 39						
P^a^	49.8%	41.11%	40.28%	25.9%	22.5%	33.7%	17.4%	31.9%	21.0%
R^b^	44.5	22.75	10.70	1.87 (< 3)		10.43		1.75 (< 3)	
KMO test	.885	.828	.840	.676		.767		.749	
Bartlett´s test of sphericity (χ^2^)	χ^2^ = 2435.72	χ^2^ = 1377.4	χ^2^ = 1478.39	χ^2^ = 853.73		χ^2^ = 1262.3		χ^2^ = 1152.6	
	sig. .000	sig. .000	sig. .000	sig. .000		sig. .000		sig. .000	

P^a^ = Percentage of variance explained by each AFE factor (eigenvalues-greater-than-one rule)

R^b^ = ratio between the difference of the first and second eigenvalues and the difference between the second and third eigenvalues

Previously, we had calculated the KMO values and the Bartlett’s test of sphericity. All the KMO test values for the six scales were greater than 0.67 and the results of the Chi-squared test in Bartlett’s test of sphericity were significant, guaranteeing the adequacy of the data set for the factor analysis that was performed (PCA). In the case of Cohesion, Flexibility, and Disengaged, the percentage of variance explained by the single factor with an eigenvalue greater than 1 was over 40%. A result that confirmed the unidimensionality of these three variables. However, two eigenvalues greater than one were identified by the PCA on the scales of Enmeshed, Chaotic, and Rigid, and the variance explained by the first factor was less than 40%. In addition, for Enmeshed and Chaotic, the quotients of the differences between the first and second eigenvalues and between the second and third eigenvalues were less than 3 in both cases (Carmines & Zeller, 1979). It may therefore be affirmed that the constructs of both Enmeshed and Chaotic as they are defined are not consistent. In the case of the Rigid scale, it was not unidimensional, although it was very close to being so. Likewise, the items with factor loadings below 0.3 in the CFA were almost exactly the same items that were not saturating the first PCA factor, in its respective scales: Enmeshed, Rigid, and Chaotic.

At this point, we decided to remove eight items from the three scales (specifically, items 10, 22, 30, and 40 from Enmeshed; 12 and 30 from Chaotic; 29 and 41 from Rigid), in order to improve the quality of the model. This decision was based on the following criteria: a) the excessively low factor loadings of the items in the CFA (all below 0.33 and six below 0.17); b) the incompliance of unidimensionality in these three constructs (the eight items removed were not saturating in the first component of their scale in the PCA, as shown in Table 2); c) the lack of convergent validity (measured with both CR and AVE); d) the deleted items were the ones that produced the highest levels of reliability on their scale –measured with Cronbach’s alpha– when they were removed from the model. Furthermore, in the three aforementioned scales, the modification indexes proposed a high number of correlations between errors, to improve the quality of the model. Thus, before dropping the items from each construct, the errors were correlated according to the modification indexes. However, the results of the model were of no better quality following this procedure, since the main problem of the model was the inadequate saturation of the items in its construct and not the possible correlation of its errors.

At a theoretical level, we proposed an explanation for the inadequate fit and the low saturation of these eight items on their respective scales. On the Chaotic scale, there was ambiguity over the significance of items 12 and 30. Item 12 (“It is hard to know who the leader is in our family”) is not necessarily an indicator of chaos within the family. Not knowing who the family leader is may mean that there is no leader or that there are two leaders with the same level of power. The sentence of item 30 (“There is no leadership in our family*”*) can imply chaos or disengagement within the family, as there is very low involvement or interaction (Olson & Gorall, 2003, Appendix 19.1).

On the Rigid scale, item 29 (“Our family becomes frustrated when there is a change in our plans or routines”) should be changed for the adolescent population, because when filling in the questionnaire, many of the participants were unaware of the meaning of “frustrate”. Item 41 (“Once a decision is made, it is very difficult to modify that decision”) has no clear meaning. Who takes the decision? Who does not permit it to be changed? Does the decision refer to rules, roles, personal issues, family issues, discipline consequences, etc.?

On the Enmeshed scale, items 4, 16, and 28 explicitly refer to extreme emotional closeness or fusion, dependency, and maximization of time spent together. However, the other four items are in a negative key and in our understanding, do not necessarily express Enmeshed, as it was defined by the authors (Olson & Gorall, 2003, Appendix 19.1). Item 22 (“Family members have little need for friends outside the family”) might also indicate lack of interest or personal apathy. Under item 10 (“Family members feel pressured to spend most free time together*”*), item 34 (*“*We resent family members doing things outside the family*”*), and item 40 (*“*Family members feel guilty if they want to spend time away from the family*”*), reference is made to negative feelings, supposedly associated with engagement, in other words, coercion in relation to item 10, jealousy under item 34, and guiltiness under item 40. However, these feelings at the stage of adolescence do not necessarily have to be linked to extremely close emotional ties or dependency on parents or siblings.

### The new FACES IV scales: convergent validity and correlation analysis

**Fig. 1:**
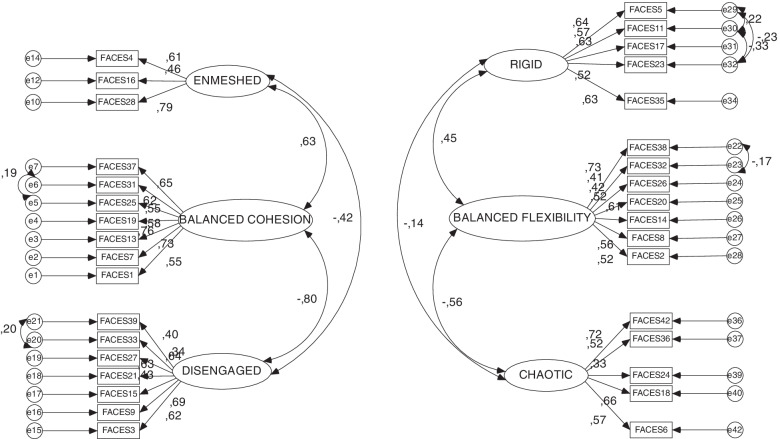
Confirmatory Factor Analysis (CFA) for the new FACES IV scales

After removing the eight items, we performed a new CFA of the FACES IV, with the 34 remaining items. The results are shown in Fig. 1.

Without the deleted items, all the new factor loadings were significantly non-zero coefficients and higher than 0.3.

After having removed the eight items specified above, the improved fit indices of the new quality model were sufficient (CMIN/df = 4.142; *p*-value = 0.000; GFI = 0.903; AGFI = 0.886; NFI = 0.835; IFI = 0.870; CFI = 0.869; RMSEA = 0.051; *p*-value of the RMSEA = 0.143), such that the adapted FACES IV instrument may be considered a good model. Three of the five values that never reached the optimum levels in the first CFA were in the new CFA within the recognized levels for a good adjustment of the model. One of the parameters that showed significant improvement was the RMSEA *p*-value (from 0.006 to 0.143), a key element to test the improved goodness of fit, because a *p*-value lower than 0.05 invalidates the quality of the model (Chen et al., 2008).

The reliability of the new constructs also increased. All the Cronbach’s alpha coefficients were above 0.7 (see Table 3), except for the Enmeshed scale, whose coefficient was not far from this value.

**Table 3:** Descriptive statistics (average scores), convergent validity, and correlation analysis for the new six FACES scales*

FACES IV scales	Mean	SD	α	CR	AVE	FACES IV Scales—Correlations				
						Balanced Cohesion	Balanced Flexibility	Disengaged	Enmeshed^a^	Rigid^a^
Balanced Cohesion	3.632	.754	.830	.826	.408					
Balanced Flexibility	3.493	.678	.730	.744	.301	.77**				
Disengaged	2.463	.723	.747	.744	.305	–.60**	–.49**			
Enmeshed^a^	2.675	.837	.655	.657	.400	.43**	.42**	–.30**		
Rigid^a^	2.827	.776	.713	.734	.357	.28**	.37**	–.07*	.32**	
Chaotic^a^	2.219	.753	.697	.701	.332	–.32**	–.36**	.52**	–.06	–.07*

(*) Cronbach’s alpha (α), CR, AVE for Convergent Validity. Pearson’s correlation matrix for Correlation Analysis. *N* = 1187

(.^a^) Adapted new scales, without deleted items

(*) *p*-value < .05

(**) *p*-value < .01

The CR and the AVE are also necessary to analyze the convergent validity (see Table 3). The CR coefficients were all over 0.7 except for the Enmeshed scale, and the AVE values were all higher than 0.3. The two scales, Rigidity and Chaotic, improved their CR by 7 and 12%, respectively and their AVE by more than 35% in both cases. Notably, both the CR and the AVE of the Enmeshed scale also improved by 26 and by 110%, respectively.

A correlation analysis was also performed, in order to evaluate the relations between the six scales (see Table 3).

The strongest correlation was between the two balanced scales, Cohesion and Flexibility, which according to Olson (2011) demonstrates concordance in the balanced family function within the zone with the healthiest function levels. There was a high negative correlation between Cohesion and Disengaged (-0.6) and a moderate one between Flexibility and Chaotic (–0.36). However, the sample presented moderate positive correlations between Cohesion and Flexibility, with both Enmeshed and Rigid.

The results were unequal for each of the two principal dimensions with regard to the non-balanced scales. Enmeshed and Disengaged showed a weak negative correlation, which makes sense, as if one increases, the other should diminish; while no significant relation was found between Rigid and Chaotic, showing the independence of both variables and that a system could be chaotic and rigid at the same time.

The important positive correlation (0.52) between Disengaged and Chaotic captures the attention, in so far as it might indicate that adolescents tend to perceive the systems where no emotional links exist as chaotic. A moderate positive correlation was also found between Rigid and Enmeshed (0.32).

### Factor analysis, validity, and reliability for the FCS and the FSS scales

A CFA was performed for each scale, FCS and FSS. The factor loadings of the items are shown in Table 4.

**Table 4:** Factor loadings for FCS and FSS scales

FCS	Factor loading	FSS	Factor loading
FCS1	.713	FSS1	.735
FCS2	.693	FSS2	.725
FCS3	.695	FSS3	.715
FCS4	.719	FSS4	.742
FCS5	.685	FSS5	.727
FCS6	.477	FSS6	.736
FCS7	.587	FSS7	.549
FCS8	.721	FSS8	.725
FCS9	.698	FSS9	.666
FCS10	.694	FSS10	.687

The factor loadings were all over 0.48 in the FCS and over 0.55 in the FSS, and most of these factor loadings on both scales surpassed the threshold of 0.7 or were very close to it.

All values of the goodness of fit indices were within the required limits (see Table 5). GFI, AGFI, NFI, IFI, and CFI were all over 0.95, which indicated an excellent fit of the model (Hair et al., 2010).

**Table 5:** Descriptive statistics (total score), AVE, CR, and the main fit indices for the FCS and the FSS scales

Scale	Mean	SD	CR	AVE	CMIN/df	GFI	AGFI	NFI	IFI	CFI	RMSEA	*p*-value
FCS	34.68	7.92	.891	.452	4.605	.982	.959	.978	.983	.983	.055	.182
FSS	37.14	7.83	.907	.494	4.144	.981	.962	.982	.987	.987	.052	.372

As was done for the six variables of the FACES IV scale, a PCA with Varimax rotation was performed for each of the FCS and the FSS scales, to test their unidimensionality. The KMO test had previously been calculated –values of 0.918 for the FCS, and 0.933 for the FSS– and the results of Bartlett’s test of sphericity (χ^2^) were statistically significant for both scales, which guaranteed satisfactory sampling adequacy and the validity of the component analysis. For each of the FCS and the FSS scales, there was only one factor with an eigenvalue greater than one –values of 5.1 and 5.6, respectively– and the variance explained by the unique factor was over 40–51.8% for the FCS and 56.4% for the FSS–, which confirmed the unidimensionality of both scales.

The Cronbach’s alpha coefficients for reliability were also excellent on both scales (0.894 for FCS and 0.913 for FSS). To complete convergent validity, we calculated CR and AVE (see Table 5), which were also within the desirable limits (CR greater than 0.8; AVE of 0.5 for FSS and slightly lower for FCS).

All these results yielded very good psychometric properties for FCS and FSS scales, confirming the robustness of their theoretical constructs.

### Correlation analysis

The correlations between the FCS and FSS scales with the other FACES scales are shown in Table 6.

**Table 6:** Correlation analysis of FSS/FCS and correlations of FACES IV with validation scales (FCS and FSS)

Scales	FSS	Cohesion	Flexibility	Disengaged	Enmeshed^a^	Rigid^a^	Chaotic^a^
FCS	.788**	.704**	.684**	–.571**	.427**	.199**	–.368**
FSS	–	.696**	.668**	–.555**	.464**	.264**	–.296**

(.^a^) Adapted new scales, without deleted items

(*) *p*-value < .05

(**) *p*-value < .01

The correlation between Family Communication and Family Satisfaction was very high (0.79), meaning that good communication in the family leads to wellbeing within the family system. The relation between these dimensions with the balanced variables was also highly positive (almost 0.7 in both cases) and followed the same trend with the non-balanced variables as were found for Balanced Cohesion and for Balanced Flexibility, which is to say, that they were negatively correlated with Disengaged and with Chaotic, and positively correlated with both Enmeshed and Rigid.

### Discriminant analysis (Predictive Validity) for the FACES IV scales

Following the methodology described by Olson (2011), a discriminant analysis for the new six FACES IV scales was performed, to determine the capacity of these scales to distinguish between problematic and non-problematic families. There were no specific or external criteria to classify problematic and non-problematic families, so these groups were defined on the basis of each person’s score on two family assessment instruments, established as valid measures of family functioning, specifically the Family Satisfaction Scale (FSS) and the Family Communication Scale (FCS). Discriminant analyses were carried out for each of the six scales of FACES IV entered as individual independent discriminators (or predictors) of the problematic/non-problematic groupings, as well as an analysis where the six scales were entered together as the independent variable.

We proceeded to define the groups, according to the individual scores on both the FCS and the FSS: first, if the score was in the top 50% of the FSS, the individual was assigned to the non-problematic group or with healthier family functioning. Conversely, if the score was in the bottom 50% of the FSS, the individual was included in the problematic group or with poorer family functioning. A similar procedure was followed by using the individual scores on the FCS. Analogous problematic and non-problematic groups were created using both the upper 40% and the lower 40% of the FSS (or the FCS) scale as their respective cut-off points. Table 7 illustrates the results of the discriminant analysis, where the four columns represent the four different ways of grouping the families.

**Table 7:** Discriminant analysis of functional/dysfunctional families (Percent Accuracy in Discriminating Groups) for FACES IV scales

	Upper versus lower 50% on FSS	Upper 40% versus lower 40% on FSS	Upper versus lower 50% on FCS	Upper 40% versus lower 40% on FCS
N for each group	Upper *N* = 575	Upper *N* = 505	Upper *N* = 599	Upper *N* = 471
	Lower *N* = 603	Lower *N* = 474	Lower *N* = 579	Lower *N* = 513
Unbalanced Scales				
Disengaged	70.5%	74.8%	71.0%	73.9%
Chaotic^a^	62.1%	65.3%	61.0%	62.1%
Enmeshed^a^	65.5%	68.0%	68.6%	66.6%
Rigid^a^	57.6%	58.5%	60.1%	61.4%
Balanced Scales				
Balanced Cohesion	75.6%	80.7%	75.6%	79.4%
Balanced Flexibility	72.4%	77.8%	74.7%	78.5%
Six scales together	77.3%	82.9%	78.4%	82.6%

(.^a^)Adapted new scales, without deleted items

GR 1 = Upper N = Non- problem Families

GR 2 = Lower N = Problem Families

The grouping method that showed the highest percentage accuracy at discriminating between the problematic and non-problematic groups was the upper 40% versus the lower 40% on the FSS validation scale. Correct placement ranged from 58.5% (Rigid) to 80.7% (Balanced Cohesion), reaching 83% when all six scales were considered together. That means that using jointly the 6 FACES variables (and employing the FSS scale as the valid criterion), the scores can predict whether a family will or will not be problematic in 83% of cases. Both Olson (2011) and Martínez-Pampliega et al. (2017) reported discriminant analyses with similar findings. Compared to the original American validation, the percentage of accuracy for our sample was lower for the Disengaged, the Chaotic, and the Cohesion scales, but higher for the Flexibility, the Enmeshed, and the Rigid Scales.

## Discussion

This paper presents an important contribution to the study of adolescent family functioning, as it is the first validation of the FACES IV package for adolescents, based on a large and heterogeneous sample, and using an elaborate sampling strategy.

The indicators of internal consistency, convergent validity, and predictive validity of the scales of the FACES IV package provided a good fit for the scales of Balanced Cohesion, Balanced Flexibility, and Disengaged and an excellent fit for Family Communication and Family Satisfaction. However, the results for convergent validity were not good for Enmeshed, Rigid, and Chaotic, which in addition never complied with the condition of unidimensionality. These three scales also showed problems for the validation of the FACES for Slovak adolescents (Sebokova et al., 2016). In the Uruguayan validation, Enmeshed and Rigid showed no fit with a single factor model, which could indicate that more than one concept is involved in these scales (Costa et al., 2013).

In our case, we decided to eliminate eight items, obtaining a reduced version of the FACES IV, but with good psychometric properties. Some of the items removed in our study also showed insufficient saturation in other European validations (Gouveia-Pereira et al., 2020; Koutra et al., 2012; Martínez-Pampliega et al., 2017; Mirnics et al., 2010) and were also almost all removed by Rivero et al. (2010) in the abridged Spanish version. In the Canadian study with adolescents (Desautels et al., 2016), saturation was very low in practically all the same items as in our work, while the items from Enmeshed and Chaotic showed acceptable or good saturation on their respective scales when the authors conducted their validation among adults. These results indicate that adolescents understand some questions differently than adults.

The confirmatory factor analysis of our final shortened version produced an acceptable model for the six scales theorized by Olson, with good reliability indices, except for the Enmeshed scale (0.66), a quite reasonable value, on the other hand, taking into account that the scale only had three items.

As regards the correlation analysis, the signs of the correlations found in our sample among the non-balanced scales of FACES IV mostly match the results from other validation studies (Baiocco et al., 2013; Costa et al., 2013; Koutra et al., 2012; Martínez-Pampliega et al., 2017; Olson, 2011; Pereira & Teixeira, 2013).

The important positive correlation found between Disengaged and Chaotic and the weak positive one between Rigid and Enmeshed are in accordance with the findings of Olson (2011) and other transcultural works. In the opinion of both Pereira and Teixeira (2013) and Mirnics et al. (2010), although the scales of Enmeshed and Rigid appear to be independent from other scales, they are inter-related.

Likewise, the high correlations within our sample between Family Satisfaction and Family Communication with the two balanced scales, as well as the high negative correlations of those four scales with Disengaged and Chaotic, are almost generalizable to all the validations that have been performed and they confirm some of the hypotheses proposed by Olson. They demonstrate that communication significantly improves family satisfaction and that cohesion and flexibility lead to healthier family systems and with more satisfactory functioning. In turn, disengaged and chaos are frequent in dysfunctional family systems.

However, unlike other studies, we found a negative correlation between Enmeshed-Disengaged and moderate positive correlations between the “healthy” dimensions (Communication, Satisfaction, Cohesion, and Flexibility) with Rigid and Enmeshed, which calls into question the familiar dysfunctionality of these two unbalanced scales. In the study by Sequeira et al. (2021), Enmeshed and Rigid seemed to be positively connected to healthy family functioning, probably due to “Portuguese cultural specificities, namely its traditional values and manifest ideological familism, that emphasizes affective closeness, explicit solidarity norms, (…) marked respect for authority and hierarchies” (Sequeira et al., 2021, p. 1660).

Everri et al. (2020) also found positive correlations between Enmeshed and Rigidity with Family Communication, Cohesion, and Flexibility in his sample of Italian adolescents. It therefore appears that Rigidity and Enmeshment are not necessarily perceived as negative by the adolescents. It may even be precisely at this stage of life where parental control and some strong emotional links are necessary for the proper development of the young person. Pereira and Teixeira (2013), Rivero et al. (2010), and Martínez-Pampliega et al. (2017) had already pointed in that direction, as they affirm that Rigid and Enmeshed appear not to be as dysfunctional as Disengaged and Chaotic, and that the families with rules and clear and strict consequences function better. Baiocco et al. (2013) also found that the younger adolescents scored higher for Enmeshed and Rigid and although the authors related it with greater family dysfunctionality, it may have more to do with the necessary parental supervision at those ages. In turn, in the studies of Everri et al., (2015, 2016), the adolescents linked Rigid to a protective and emotional tie, related with greater interest and parental commitment and deduced from their results that Rigid is not negative per se, but that it depends on the positive or negative dimensions of family functioning with which it is associated.

Olson (2008) had previously noted that the non-balanced variables were not necessarily always dysfunctional and that the time of the life cycle, along with other cultural and religious aspects, should be also considered when interpreting these two variables. It is important to note that almost all the studies where the behavior of the variables Enmeshed and Rigidity was unexpected, have been carried out in Mediterranean cultures (Portugal, Spain, Italy, and Greece). Enmeshed families and rigidity are perhaps viewed within those family environments as part of the culture and are even considered desirable for acceptable functioning of a family.

In conclusion, the results of our study corroborate the factorial structure of the circumplex model proposed by Olson (2011) and demonstrate the transcultural applicability of FACES IV, FCS and FSS questionnaires. The version validated in this work (reduced for Enmeshed, Rigid, and Chaotic) presents the FACES IV package as a valid and useful instrument for the assessment of family functioning of Spanish adolescents.

Moreover, in the sample of Spanish adolescents, the scale of Family Communication, the scale of Family Satisfaction, and the scales of Balanced Cohesion, Balanced Flexibility, Disengaged and Chaotic of FACES IV function in the way that was anticipated through the Circumplex Model, and that has been confirmed in all the validations with the adult population. However, the scales Enmeshed and Rigid work differently in adolescents than they do in adults, because both scales are not always perceived as dysfunctional by adolescents. On the contrary, these two variables are frequently considered positive among adolescents and are associated with greater family emotional attachment, flexibility, communication and satisfaction.

### Limitations of the study

The strengths of the present study are the size of the sample and its variability. However, the conclusions were based on the perceptions of adolescents, without any information provided by other family members, which might give it a certain bias.

Additionally, as the six FACES scales have a different number of items, it is not possible to calculate the ratio scores. It would therefore be recommendable to substitute the items that have been removed or to adapt them to the adolescent vocabulary and to the specific requirements of this stage of the life cycle.

In future investigations, further studies should be performed on the Enmeshed scale, in order to improve its consistency and reliability. Likewise, additional studies will be necessary to confirm the trend observed for Enmeshed and Rigid scales among adolescents.

## Data Availability

The datasets generated and analysed during the current study are available in the Harvard Dataverse repository, https://dataverse.harvard.edu/dataset.xhtml?persistentId=doi:10.7910/DVN/UA5GTO.
